# Phenotypes, endotypes and genotypes of atopic dermatitis and allergy in populations of African ancestry on the continent and diaspora

**DOI:** 10.3389/falgy.2023.1203304

**Published:** 2024-01-24

**Authors:** N. Lunjani, T. Kerbelker, F. B. Mdletshe, C. Hlela, L. O’Mahony

**Affiliations:** ^1^ APC Microbiome Ireland, University College Cork, Cork, Ireland; ^2^ Division of Dermatology, University of Cape Town, Cape Town, South Africa; ^3^ Department of Peadiatrics, University of Cape Town, Cape Town, South Africa; ^4^ Division of Otorhinolaryngology, University of Witwatersrand, Johannesburg, South Africa; ^5^ Department of Medicine, University College Cork, Cork, Ireland; ^6^ School of Microbiology, University College Cork, Cork, Ireland

**Keywords:** atopic dermatitis, microbiome, immune mechanism, skin barrier disruption, atopic dermatitis in African populations

## Abstract

Atopic dermatitis is a complex inflammatory condition characterized by synergist interactions between epidermal and immune related genotypes, skin barrier defects and immune dysregulation as well as microbial dysbiosis. Ethnicity-specific variations in clinical presentation, immune endotypes and genetic susceptibility have been described in diverse populations. We summarize available data with specific consideration of AD in populations of African ancestry. Some highlights include the observation of AD lesions on extensor surfaces, lichen planus-like AD, prurigo type AD and follicular AD in African populations. In addition, a consistent absence of dominant filaggrin gene defects has been reported. The detection of normal filaggrin protein content in AD skin implicates the contribution of alternative mechanisms in the pathogenesis of AD in African patients. Markedly high IgE has been described in paediatric and adult African AD. While Th2, Th22 and Th17 activation in African AD skin shares the same direction as with other populations, it has been noted that the magnitude of activation is dissimilar. Reduced Th17 cytokines have been observed in the circulation of moderate to severe paediatric AD.

## Graphical Abstract

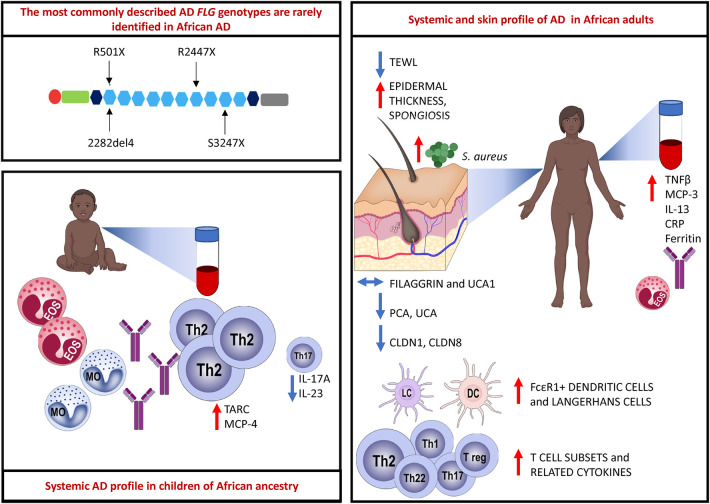

The most commonly described *FLG* loss-of-function (LoF) mutations located in the epidermal differentiation complex (EDC) on chromosome 1q21 are rarely identified in African AD. Paediatric AD is characterized by an abundant Th2-related cytokine milieu, and reduced Th17-related cytokines have been observed. Eosinophils, monocytes and total and specific IgE are elevated in the circulation of children with AD. There is a multipolar cytokine profile in adult AD with increased eosinophils and total IgE also noted. Adult AD skin biopsy samples show increased epidermal thickness and spongiosis. The content of filaggrin protein and filaggrin breakdown products such as urocanic acid-1 (UCA1) are unchanged in African AD skin. Filaggrin breakdown products such as pyrrolidone-5-carboxylic acid (PCA) and urocanic acid (UCA) are reduced. Tight junction proteins such as claudin-1 (CLDN1) and claudin-8 (CLDN8) expression is reduced in AD in adult Africans. There is increased influx of Fc*ε*R1 bearing dendritic cells and Langerhans cells as well as increased expression of diverse T cell subsets and related cytokines. *Created with* BioRender.com.

## Introduction

Atopic dermatitis (AD) is a highly complex multifactorial inflammatory skin disease that follows a relapsing-remitting chronic course (1). AD disproportionately affects children globally (2) and its incidence has increased rapidly during recent years in African countries (3, 4). Current data from South Africa as an example suggests that the changing diversity of environmental exposures, socioeconomic factors, diet, and lifestyles across African societies might provide unique disease promoting effects in early life, in populations where a low risk of developing AD and allergic disease was previously documented (4).

### Features of atopic dermatitis

The hallmark features of AD are well established. It is characterized by intense pruritus (2). Notable skin lesions at predilection sites such as the flexural surfaces include xerosis, erythema, oedema, excoriations, oozing, crusting and lichenification. A wide spectrum of clinical AD phenotypes that vary with age and ethnicity are also key clinical features (5). Atopic dermatitis is thought to arise from gene-gene interactions and gene-environment interactions that impact the epidermal barrier and host immunity (6, 7). The increasing prevalence of the disease supports the role of environmental factors interacting with host immune responses, microbial composition, and epidermal barrier factors in the pathogenesis of AD (6, 7).

Pruritus responses may be more intense in patients of African descent (8). Larger mast cell granules and differences in the subgranular structure, and localization of the proteases, tryptase and cathepsin G in the mast cells of African individuals compared to Europeans, may account for this (9). Other mechanisms of itch may account for differences in itch perception among different ethnicities however these have not been adequately tested (8, 9).

**Figure 1:**
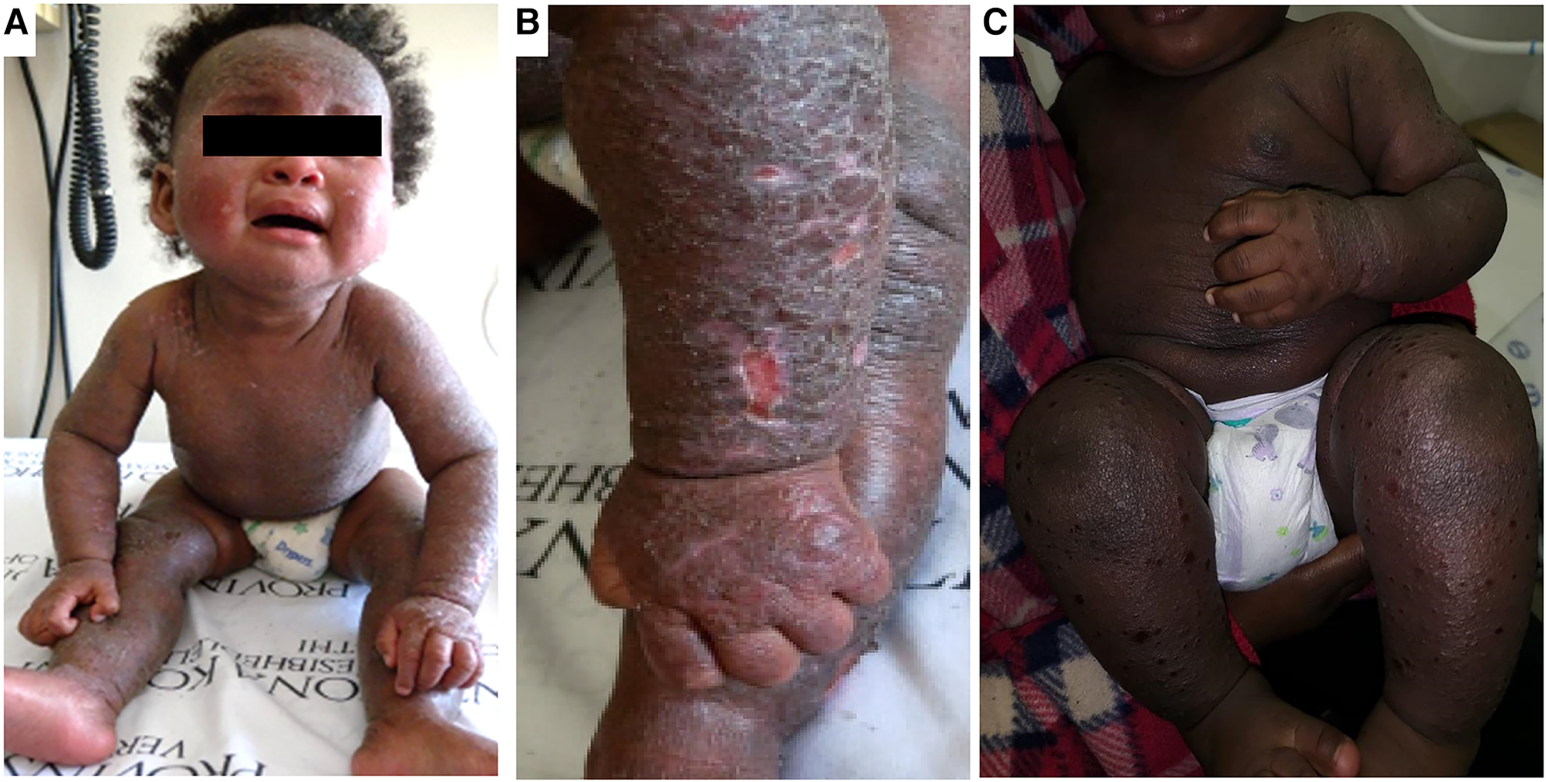
Clinical aspects of atopic dermatitis in infancy. Panels (**A,B**) shows an infant with marked lichenification of the skin with erythroderma and hyper accentuation of skin markings. There are also areas of hyperpigmentation. Panel (**C**) shows another infant with extensive erythroderma and well demarcated shallow ulcerative lesions of eczema coxsackium.

**Figure 2:**
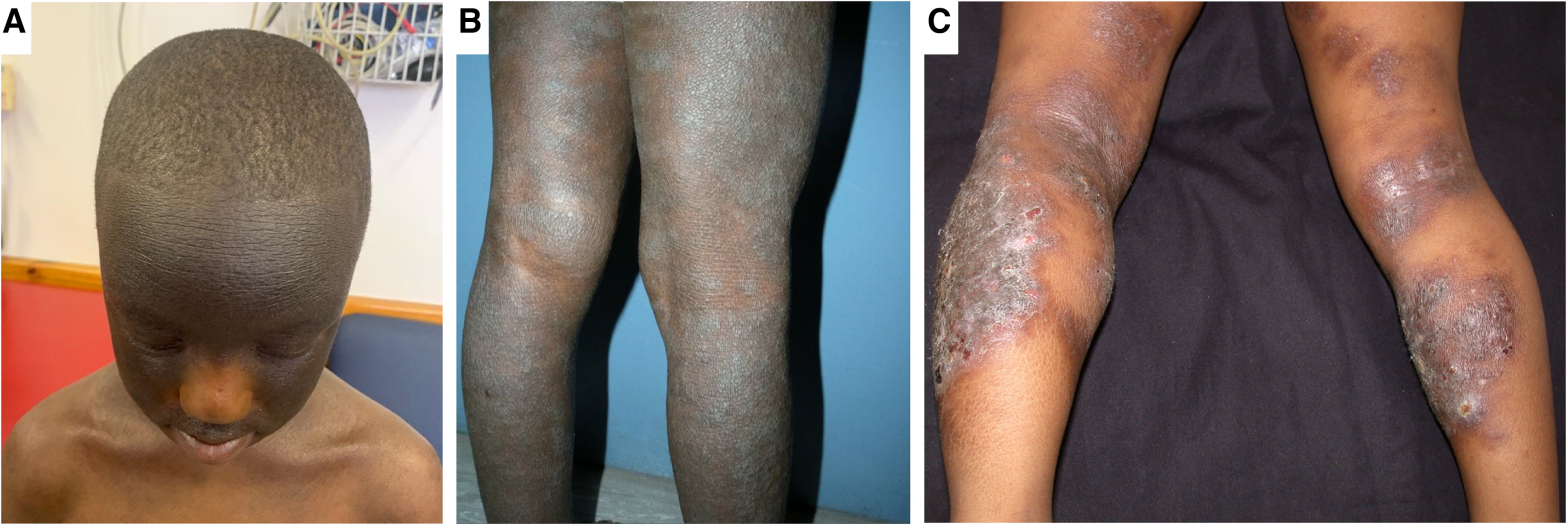
Clinical aspects of atopic dermatitis in childhood. Panel (**A**) shows marked hyperpigmentation of the face and hyper accentuation of skin markings with sparing of the central face. This patient does not fully typify the central facial sparing as the sparing is limited to the nose. Panel (**B**) shows lichenification, in addition to hyperpigmentation and dryness of the legs. Panel (**C**) shows eczematous plaques of eczema in both extensors and flexors with superimposed crusting in an older child.

**Figure 3:**
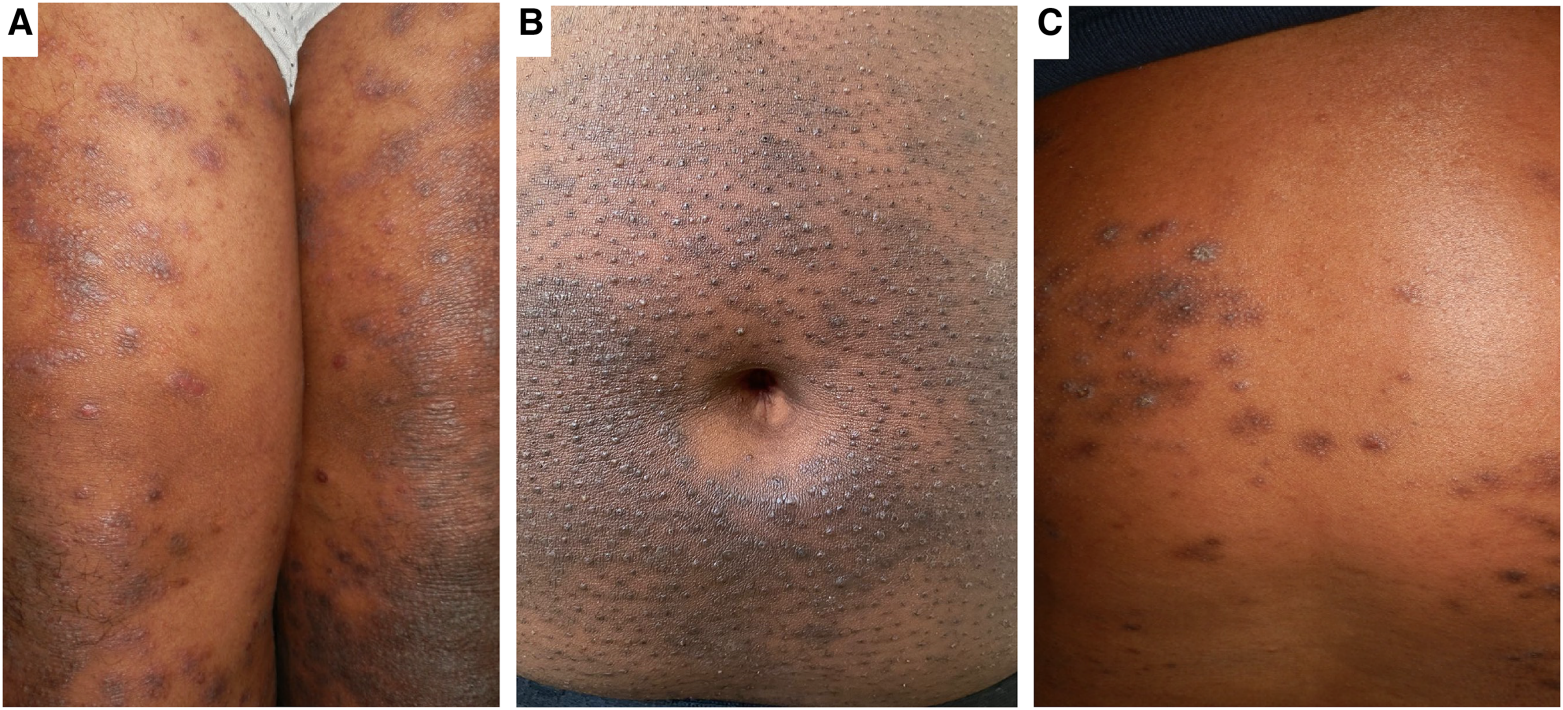
Clinical phenotypes of AD in African populations. Panel (**A**) shows Lichen-Planus like AD with hyperpigmented flat topped papules with shiny surface distributed over the thighs. Panel (**B**) shows hyperpigmentation and densely aggregated follicular papules (folliculocentric) characteristic of the follicular AD phenotype on the abdominal wall. Panel (**C**) shows the prurigo-like AD phenotype characterised by dome shaped papules with hyperpigmented areas.

Morphological variations in AD phenotype have been noted with increased frequency in individuals of African and Asian ancestry (5, 7). AD patients of Asian ancestry may present with parakeratosis which is the incomplete maturation of keratinocytes and the retention of nuclei in the stratum corneum (10). It is an atypical morphological feature of AD and is more consistent with psoriasis (10). It has been observed that African individuals may have a pronounced treatment-resistant lichenified type and often present with severe forms of AD (Figure 1) (11). The follicular type variant characterised by densely aggregated follicular papules, has been described in darkly pigmented skin (Figure 3B) (5, 7, 9). Long-standing disease may present with prurigo AD type (Figure 3C) (8). Perifollicular accentuation, papulation, scaling, lichenification, and pigmentary changes may also be more prominent in patients with darker skin (8). Transepidermal water loss (TEWL) is notably higher in individuals of African descent compared to European descent due to the lower ceramide content in the skin in addition to several other more granular phenotypic characteristics (Table 1) (8, 12–16). Clinical variations in AD phenotypes are also age-dependent (Figures 1–3) (5, 11).

**Table 1:** Molecular skin phenotype of AD in African populations.

Author	Study type	Participants	Method	Key findings
Lang et al. (12)	Case-Control study	Tanzanian Adults with AD (*n *= 10) Controls (*n *= 10)	RNA Seq skin biopsy samples	FLG; LOR; PSORS1C2; sciellin; LCE genes were not downregulated in AD skin lesions compared to healthy skin and FLG breakdown products such as UCA1; UROC1; OPLAH; GGCT; ARG; CASP14; TYR were comparable between AD and healthy skin. Significant downregulation of tight junction genes such as CLDN1; CLDN8; CLDN10 and adherens junction genes CDH10; CDH12; CDH19; CDH20 and keratin genes KRT33A; KRT77; KRT79 Significant downregulation of lipid metabolism genes HAO2; ELOVL3; GAL; FAR2; AWAT1; DGAT2; FABP7; FADS1 and 2.
Wongvibulsin et al. (13)	Case-Control Study	African American with AD (*n *= 6) Controls (*n *= 6)	Histology of skin biopsy samples	Psoriasiform and spongiotic dermatitis in lesional skin Significantly greater epidermal thickness of lesional skin compared to healthy skin
Biagini et al. (14)	AD profiling	African American with AD (*n *= 410)	Keratinocyte FLG levels TEWL	Significantly higher FLG expression in non-lesional skin of African Americans compared to European Americans non-lesional skin TEWL was significantly lower in African American lesional and non- lesional skin
Sanyal et al. (15)	Case-Control Study	African American adults with AD (*n *= 15) Controls (*n *= 9)	RNA Seq (skin)	KRT16; ELOVL3; FABP7; FADS1/2; LOR; FLG2; CLDN3/8/10/16/23 genes were downregulated in AD FLG and OVOL1 genes were not downregulated as observed in European AD
Thawer-Esmail et al. (16)	Case-Control study	South African AmaXhosa with AD (*n *= 69) Controls 81)	HPLC Liquid chromatography-tandem mass spectrometry	Reduced FLG degradation products such as PCA; UCA; FAA; FAA and derivatives are reduced in AD skin compared to healthy skin

### Diagnostic criteria and disease severity scoring of AD

Clinical AD diagnostic criteria have not been adequately evaluated in African populations in the developed and developing settings (17). The Hanifin and Rajka criteria is the earliest formal diagnostic criteria (18). It has been distilled into the UK Working Party diagnostic criteria which is widely used especially in epidemiology research (17). However, the UK Working Party was a poor predictor of AD in a community-based validation study in South Africa suggesting that it may not accurately identify cases of African AD (19). There is a general consensus amongst key stakeholders in AD care that AD severity scoring indices are important tools in AD management (20). However, these indices or outcomes measures also have limited application in darkly pigmented skin as they are reliant on the presence of erythema (21). Erythema is not readily appreciable in darkly pigmented skin, and this leads to gross underestimation of disease activity and severity (21, 22). This situation is not mitigated by assessments conducted by well experienced dermatologists who predominantly treat patients with darkly pigmented skin types (23). Recently the Patient-Oriented SCORing Atopic Dermatitis (PO-SCORAD) has been validated for evaluating AD severity in a multicenter study conducted in seven African countries (24). This subjective tool is useful for monitoring disease activity between physician consultations and guiding treatment strategies (24). Nevertheless, objective biomarkers of disease and disease severity are desirable to accurately diagnose, guide clinical management and monitor response to novel treatments in clinical trials.

### Prevalence of atopic dermatitis

This debilitating dermatosis affects up to 30% of children and up to 10% of adults throughout the world (2). 1%–3% of the elderly population in industrialised countries has AD with a 2:1 male predominance observed (25). Globally the prevalence of AD has been on the rise parallel to industrialization with 2–3-fold increase over the past few decades (1). The increasing trend continues in low-middle income countries (1). Onset of disease is commonly between 3 and 6 months of age (2). The International Study of Asthma and Allergies in Childhood (ISAAC) produced the most robust global epidemiology data for children and adolescents (3). ISAAC revealed that the prevalence of AD varies greatly throughout the world with the highest prevalence (15%) of AD in both 6–7-year-olds and 13–14-year-olds reported for urban Africa, the Baltics, Australasia, and Northern and Western Europe (3). An overall female: male ratio of 1.3:1 was also reported, consistent with findings elsewhere (3).

The high urban prevalence is consistent with observations made in South Africa showing an urban-rural gradient characterized by high allergic sensitization and allergic disease in urban settings compared to rural communities of the same ethno-linguistic background (4). A growing number of reports even dating back to the mid-90s already identified a higher prevalence of AD in populations of African ancestry in the UK (26). More recent data, from the US and UK also report an increasing prevalence and a significantly higher risk of severe forms of AD and asthma in individuals of African ancestry compared with those of European descent (22, 26–30). Based on ISAAC phase I and III data AD prevalence in Latin America ranges from 4% to 25% (3). A positive association between African genetic admixture (independent of socioeconomic factors) and atopy and asthma has been noted in some Latin American countries where African ancestry make up a significant composition of the population (31).

### Environmental risk factors

The increased prevalence of AD in developed or urbanised settings highlights the role of the environmental exposures in altering disease risk (1). A wealth of literature has drawn parallels between the increase in respiratory allergy and environmental factors specifically ambient air pollutants associated with urbanization (32). However, fewer studies examined the role of environmental pollutants on allergic disease in the skin. Some of these show that air pollution, characteristic of urbanization, positively associates with AD prevalence (33, 34). Prenatal exposure to certain air pollutants may link to allergy development through dysregulated immune responses mediated by microRNA and DNA methylation (34). Birth cohort studies suggest that indoor and outdoor air pollution associates with AD development (33, 35). Longitudinal studies also suggest that various air pollutants associate with AD exacerbations and disease persistence (34). Environmental exposures such as polluted air and maternal cigarette smoking have been shown to exert their effects on the epigenome to influence AD risk (34, 35). The role of environmental pollutants in AD pathogenesis is supported by the higher prevalence of disease in urban settings. Children living in urban centres of Latin America as an example, have been shown to have a much higher prevalence of AD than their rural counterparts (36). Traffic related air pollutants (TRAP), volatile organic compounds (VOCs) and second-hand smoke (SHS) exposure increases the risk of atopic dermatitis and other atopic diseases in children including asthma (32–38). These pollutants induce oxidative stress to protein and lipids resulting in epidermal barrier disruption and triggering an inflammatory cascade (32, 34, 35, 39). Itching and scratching is also elicited following oxidative stress in animal models (40). Environmental oxidants such as air pollutants and solar radiation were shown to cause oxidative protein damage in the stratum corneum (41). It is anticipated that this scenario can be much worse in regions that experience more sunlight and high temperatures which transform air pollutants and particulate matter. Increased IL-4 producing T cells were observed in children following VOC exposure (42). Exposure to tobacco smoke was associated with hypomethylation of thymic stromal lymphopoietin (TSLP) 5′ CpG island leading to increased TSLP expression and AD development (43). The impact of these environmental exposures and others on allergy development is complex and variable, likely reflecting differences in timing and duration of exposure, dose of exposure and host genetic and immune-mediated susceptibility factors.

Research within South Africa demonstrates a lower overall prevalence of atopy in rural areas compared with urban areas over the decades (4), which highlights the importance of environmental factors on allergy risk or protection, but this rural-urban gradient is rapidly diminishing over time (4). While allergy rates have been shown to be different between South African rural and urban settings, allergic diseases still do arise in both these diverse environments. The farming environment has been shown to have generally protective effects in other parts of the world, potentially due to exposure to a more microbially diverse environment (44–46). In addition, a recent intervention study in an urban daycare centre modified its outdoor environment to include sod, forest floor segments and planters. They observed modification in skin and gut microbial composition which coincided with an increased tolerogenic immune profile (47).

### Genetic risk factors

Discovery of the association between filaggrin gene mutations with epidermal disruption in AD has improved our understanding of the disease (48). However, this genotype plays a role only in a minority of patients. The important role of loss-of-function (LoF) mutations and copy number variations in the FLG gene located within the epidermal differentiation complex (EDC) on chromosome 1q21 and AD risk was first documented in 2006 (48). However, FLG mutations are not uniformly distributed globally. p.R501X and c.2282del4 are commonly described variants in European populations (49) and have not been shown to predominate in African populations (Table 2) (16, 48, 50–66).

**Table 2:** AD genotypes in populations of African ancestry.

Author	Sequencing method	Participants sample size (*n*)	Key findings
Berna et al. (50)	Massively Parallel Sequencing (MPS)	African American with AD *n *= 326	13 uncommon *FLG2* alleles associate with increased AD remission
Fulton et al. (51)	Array based sequencing	African American with AD *n* = 588	AA participants with AD were more likely to have the CNV20 *FLG* repeats that European Americans with AD, however these differences do not associate with AD risk.
Margolis et al. (52)	NGS	African American with AD *n *= 236 AD; 160 controls	CNV associates with AD, but independent of *FLG* LoF mutations
Margolis et al. (53)	MPS	African American with AD *n* = 326	Many population specific *FLG* LoF variants are observed in African Americans whereas 4 variants predominate in European populations
Almoguera et al. (54)	GWAS	African American with AD *n *= 2420 AD; 3,423 controls	rs3811419 risk allele could be associated with increased expression of FLG antisense RNA 1 (FLG AS1) which could reduce *FLG* expression
Mathyer et al. (55)	Array based sequencing	African American with AD *n *= 39	Novel *FLG* LoF such as c.488del G; p.S3101 identified and p.R501; p.R826 replicated. p.S3316 is likely an African ancestral *FLG* LoF
Margolis et al. (56)	MPS	African American with AD *n *= 262	Exon 3 *FLG* LoF variants p.R501X; p.S3316; p.R826X. Uncommon *FLG* variants associate with persistent AD. No singular LoF variant predominates in this population
Fernandez et al. (57)	Long range PCR	Ethiopian with AD *n *= 105	No association between AD disease severity and *FLG* CNV
Quiggle et al. (58)	Genotyping of *FLG* CNV and 2 common European variants	African American with AD *n *= 37	Low CNV associates with moderate-severe AD
Taylan et al. (59)	Whole exome sequencing TaqMan assays	Ethiopian with ichthyosis vulgaris (IV) and AD *n *= 22 *n *= 155 IV/AD *n *= 192 controls	No *FLG* LoF mutations identified. Several EDC (*FLG2* S2377X; *TCHH S207X; TCHHL1* Q294X *and* non-EDC related variants detected
Polcari et al. (60)	PCR *FLG* exon 3	African American with AD/IV *n *= 18 AD; *n *= 17 controls	4/18 AD participants had R501X (*n *= 1); 2282del4 (*n *= 2); R826X (*n *= 1) and 1/17 controls had R826X mutation
Thawer-Esmail et al. (16)	Long range PCR complete *FLG* exon 3 sequencing Taqman allelic discrimination assay	South African AmaXhosa with AD *n *= 69 with AD (31 with features of IV) *n *= 81 controls	All participant samples were wildtype for R501X; 2282del4; R2447X; S3247X mutations
Margolis et al., (61)	Whole exome sequencing Taqman allelic discrimination assay	African American with AD *n *= 60 (WES) *n *= 100	Sequencing of S100 fused-type protein (SFTP) identified *FLG* Q501X; R3409X; S3707X LoF mutations; *FLG2* S2392X; S2377X LoF mutations and *TCHHL1* Q294X. All but S2377X had a low MAF.
Margolis et al., (62)	Whole exome sequencing Taqman assay	African American with AD *n *= 299	*FLG2* S2377X; H1249R mutations are associated with persistent AD
Margolis et al. (63)	Taqman allelic discrimination assay	African American with AD *n *= 370	Common *FLG mutations* R501X; 2282del4; R2447X; S3247X are rare in African American participants but when present associate with persistent AD
Winge et al. (64)	PCR Taqman assay	Ethiopians with AD/IV *n *= 103 AD *n *= 7 IV *n *= 110 controls	No common *FLG* LoF mutations were detected. A novel *FLG* 632del2 mutation was identified in 1 AD participant
Gao et al. (65)	Taqman genotyping BeadXpress genotyping	African American with AD *n *= 187 AD (32 with ADEH) *n* = 152 *n *= 177 reference	6/188 AD participants were heterozygous for R501X mutation. This mutation had a stronger association with ADEH (atopic dermatitis eczema herpeticum) 2282del4 mutation was not detected in AD or ADEH rs12730241 and rs2065956 *FLG* SNPs were associated with increased AD/ADEH risk
Howell et al. (66)	Genotype R501X; 2282del4	African American with AD *n *= 12 moderate AD *n *= 11 controls	All participants had a wildtype *FLG* genotype for these mutations
Palmer et al. (48)	Taqman assay	North African *n *= 124 healthy	R501X allele frequency was not detected in the 124 participants screened. 2282del4 allele frequency was not detected in 121 samples screened

Recent studies have suggested that polymorphisms in other barrier associated genes, such as Filaggrin-2 gene variants (62) and uncommon FLG exon 3 loss-of function (LoF) variants have been described in a minority of individuals of African ancestry and are thought to collectively confer a more persistent disease phenotype (56). Ethno-specific mutations have been reported in Japanese, Chinese, Korean and Bangladeshi populations that associate with specific phenotypes such as dryness (67–69). In particular the c.3321delA is specific to Asian population and is the most common variant in the Chinese Han population (69). A few studies have been conducted but were unable to reproduce a strong AD link to these specific variants in individuals of African ancestry (Table 2) (16, 48, 50–66).

FLG intragenic copy number variation (CNV) impact gene dosage and consequently the amount of filaggrin produced in the epidermis (70). CNV has been shown to associate with AD risk in a large study of Irish paediatric AD (70). whereas CNV in Ethiopian AD was not associated with AD disease severity (57). CNV vary with ethnicity however this difference did not associate with AD risk in a study including African American and European American participants (58). Thus far, to our knowledge, CNV have not been sufficiently studied in African ancestry and have not shown an association with AD in the few studies reported on African populations (57, 58).

Genome-wide association studies (GWAS) analysis identified over 30 further genetic risk loci including FLG (71). Notably, IL-13 also demonstrated the most significant association with AD among the susceptible gene loci identified. In addition, some of the susceptibility loci overlap with known asthma risk loci, highlighting shared “atopy” risk. Novel risk loci identified were related to immune regulation (71). Other AD associated polymorphisms occur in genes encoding elements of innate immune signaling e.g., TLR-2 and TLR-4 as well as T-cell immunity (72). Variants of Th2 cytokine genes (IL-4; IL-5; IL-9; IL-13 including RAD50) found on chromosome 5q31-33 associating with AD have been described but need confirmation in large studies (73–75). These observations underscore the important role of immune mediated pathogenesis of AD. This accumulating evidence supports the robust contribution of factors other than epidermal barrier defects in the pathogenesis of AD, such as a Th2 predominant multipolar immune activation as this can deplete epidermal barrier proteins and tight junction protein expression (75–79).

### Immune mechanisms in AD

Innate and adaptive immune responses contribute to AD pathogenesis. Polymorphisms in Pattern Recognition Receptors (PRRs) such as Toll-Like Receptor 2 (TLR2) - the main receptor responsible for recognizing staphylococcal ligands, Nucleotide - binding oligomerization domains 1 (NOD1) and NOD2 are associated with AD in a way that enables *S. aureus* overgrowth (80). Low levels of CD14, a multifunctional receptor that induces innate immune cell activation via TLR dependent pathway have been reported in atopic children (81). Keratinocytes, sebocytes, eccrine glands and mast cells contribute to innate immune function by producing antimicrobial peptides (AMPs) such as cathelicidin (LL-37), human beta defensins (hBD2; hBD3) and dermcidin to protect the host from infection (82, 83). Levels of multiple AMPs are reduced in AD patients and have defective antimicrobial function. This likely enables *S. aureus* colonization as it has been observed that over 90% of AD patients exhibit *S. aureus* colonization in lesional and non-lesional skin (44).

T cell infiltration is well documented in AD and accompanies the histological features of spongiosis (7). In recent years there has been a growing appreciation that in addition to dominant Th2 immune responses, there is multipolar immune activation that include Th1 (IFNγ), Th17 (IL-17A; IL-23) and Th22 (IL-22) activation (7). These immune pathways correspond with particular features (5, 10, 11). Th1 activation has been described in chronic AD lesions, Th17 activation has been described as feature of Asian AD that corresponds with psoriasiform features in AD, Th22 activation has been demonstrated in epidermal hyperplasia of lichenification (5, 10, 11). Interestingly, prurigo is often a co-occurring phenotype in AD especially in African AD, however the immune activation of prurigo nodularis in African skin feature Th22 immune responses with no Th2 activation-a departure from prurigo observed in European patients (84). There is a dearth of literature providing detailed data on immune mechanisms of AD in populations of African ancestry globally, nevertheless a handful of studies in African adults and children provide some evidence of differential host immune responses in this disease phenotype (Table 3) (12, 13, 15, 84–87). It is important to describe immune pathways activated in AD patients of African ancestry to determine the potential efficacy of currently available biologics targeting specific pathway in this demographic.

**Table 3:** Immune endotypes of AD in African populations.

Author	Study type	Participants	Method	Key findings
Lunjani et al. (85)	Case-Control Study	South African AmaXhosa Children (1–3 years) With AD (*n *= 79) Controls (*n *= 71)	RNASeq of Peripheral Blood Mononuclear (PBMC)	Distinct PBMC gene expression patterns were observed in children with AD. 132 differentially expressed genes were identified with *IGHE* being the most highly upregulated gene. Pathway enrichment analysis identified a range of lymphocyte patterns-Th1;Th2; Th17 and innate signalling patterns eg. TLR signalling, that were downregulated in AD compared to healthy volunteers.
Lunjani et al. (86)	Case-Control Study	South African AmaXhosa Children (1–3 years) with AD (*n *= 78). Controls (*n *= 74)	Plasma sample analysis with MSD quickplex	Significantly elevated Th2 related cellular responses, cytokine, and chemokines such as eosinophils, monocytes, TARC, MCP-4, IL-16, total IgE and allergen specific IgE. Urban subgroup with elevated IL-4 correlating with multipolar cytokine production. Notably reduced Th17 related cytokines such as IL-17A and IL-23 was also detected.
Lang et al. (12)	Case-Control study	Tanzanian Adults with AD (*n *= 10) Controls (*n *= 10)	RNA Sequencing of skin biopsy samples RT-PCR	Th2 cytokines (IL-13; IL-4R; CCL-13; CCL-17; CCL-18; CCL-26); Th22 cytokines (IL-22; S100As); Th17 (IL-17A; IL-23A; IL-12; PI3; DEFB4B and Th1 cytokines (IFN*γ*; CXCL9; CXCL10; CXCL11 were overexpressed in AD skin including IL-21
Sims et al. (87)	AD profiling	African American Adults with AD *n *= 25	Serum Olink multiplex assay and Luminex bead based multiplex immunoassay	African American AD was associated with higher EASI scores and high inflammatory profile characterized by TNFβ; MCP-3; IL-13 among other inflammatory biomarkers
Wongvibulsin et al. (13)	Case-Control study	African American Adults with AD (*n *= 6) Controls (*n* = 6)	Systemic laboratory inflammatory markers RNA Seq skin biopsy samples	Th1 (IRF1); Th2 (IL-4R, CCL18, CCL26); Th17 (IL-17A, IL-23A) and Th22 (IL-22) related genes and many others were upregulated in AD Inflammatory markers such as CRP; Ferritin and eosinophils were elevated in African American AD compared to healthy controls and European American AD
Belzberg et al. (84)	Case-Control study	African American Adults with prurigo nodularis (*n *= 10) and controls (*n *= 10)	Immunohistochemistry and RNA Seq (skin) Flow cytometry (PBMC)	Th22/IL-22 axis was upregulated in the systemic circulation and lesional skin of PN. Greater Th2 related cytokine (IL-4, IL-5 and IL-13) association with PN in European Americans and not AA.
Sanyal et al. (15)	Case-Control Study	African American Adults with AD (*n *= 15) Controls (*n *= 9)	Circulating total IgE Immunohistochemistry staining of skin biopsy samples RNA Seq (skin) RT-PCR (skin)	Elevated total IgE (compared to European AD). Increased infiltration of Fc*ε*R1+DCs and Langerhans cells Th1; Th2; TH17 and Th22 related genes including IL-9 and FoxP3+ Tregs were upregulated in lesional skin compared to non-lesional skin. IL-10; CCL-13; TSLP were exclusively upregulated in African American AD (compared to European American AD).

### Atopic dermatitis and the Th2 axis

AD is often associated with elevated serum immunoglobulin (IgE) and considered a foreshadow of the atopic diathesis - IgE-mediated food allergy, asthma and allergic rhinitis (6). Moderate to severe AD carries a higher risk of developing atopic co-morbidities. About 20%–30% of infants follow this disease course. Additionally, AD susceptibility loci increase the risk of the atopic march (88). Notably this group of children did not outgrow AD. It has recently been reported that children with AD who progress along the atopic march have a 2.7-fold increased risk of neurodevelopmental disorders (89). African populations are thought to represent a high IgE producer genotype (90–93). There is a strong association between IgE mediated sensitization to food allergens, aeroallergens and AD but the functional mechanisms of IgE in AD needs further clarification (2, 94). South African children for example have similar IgE-mediated food allergy rates as described in industrialized countries (4) however it has been noted that African children display population specific IgE sensitization characterized by large positive food allergen skin prick test (SPT) wheals and high ImmunoCAP IgE levels with no clinically relevant food allergy (i.e., Allergen tolerance) and distinct atopic march trajectories have been observed in children of African ancestry (95–98). These population-specific IgE-related observations have been confirmed in children with low detection of cross-reactive carbohydrate determinants (CCD) (85, 86) in direct contrast to the common argument that high total and specific IgE in the tropics/subtropics reflect endemic helminth infection. Furthermore African-specific allele frequencies that amplify Th2 immune responses have been identified (91–93). This may underpin the increased allergy risk. *IL-10* and *IL-4* polymorphisms that associate with allergic disease in African children have also been observed (92).

Recently, we described an AD immune endotype in African children with moderate to severe AD that was characterized by high circulating Th2 related cytokines and chemokines TARC, MCP-4, IL-16 and low Th17 related cytokines IL-17A and IL-23 (86). Sanyal et al. described high amplitude of total IgE in adult African Americans compared to European Americans (15). Furthermore, they noted that Th17 related cytokines were elevated in the skin of African American AD compared to African American healthy counterparts but not to the same magnitude as European AD. Differences in paediatric and adult AD have also been described, mainly characterized by similar polarization of Th2 cells (5). IL22/Th22 expression and Th17 subsets, which contribute to disease chronicity, exhibited lower expression in children compared to adults. Absence of Th1 activation in paediatric AD compared to adult AD has also been documented (5, 11).

### The atopic march

The atopic march refers to the sequential progression to allergic rhinitis, food allergy or asthma with AD as the entry point. It is thought to arise from shared atopic genetic predisposition, immunological responses, and environmental risk factors (6). In an American study of 18,596 AD participants where 51% were African American, it was observed that African American children had an atopic trajectory that was characterized by progression from AD to asthma whereas European American children progressed from AD to allergic rhinitis and the AD -IgE mediated food allergy trajectory was predominant amongst Asian children (98). A smaller study (*n* = 601) reproduced the “AD to asthma” atopic trajectory observed in African American children (14). Furthermore, they determined that the observed asthma risk in African American children was six times higher than European American children. This was associated with higher reports of parental AD, parental asthma and increased exposure to environmental pollutants such as secondhand smoke and traffic related air pollution (14, 98). Region-specific variations in allergic disease presentation have been reported. Colombian researchers have observed that the clinical presentation of allergy in their region is not consistent with the putative “atopic march”, but the natural history of allergy is skewed towards a debut with respiratory symptoms (99). This is possibly influenced by the tropical climate, which supports high mold, cockroach, and house dust mite allergen load in humid conditions. It is important to note that the Colombian population has a significant population of African descent, and this genetic background may have distinct clinical presentation in this regional environment (25). It has also been noted that urbanization in developing countries often occurs in the context of economic deprivation and this promotes exposure to noxious substances that might influence immune function and modify allergy susceptibility (25, 99).

### Microbiome disruption in AD

Diverse communities of commensal microbes on internal and external body surfaces play essential roles in regulation of host metabolic responses, epithelial barrier function, immune education, and immune regulation. Within the gut, individual microbes, microbial components and individual metabolites (e.g., short-chain fatty acids, histamine) are continuously being newly described that influence host immune responses, including those that impact disease processes within the skin (100–102). Associations between AD risk and gut microbiota composition and metabolism are evident across multiple studies (103), but it is still unknown if gut microbiota alterations are a cause or consequence of AD. Importantly, different microbial strains can dominate in different populations across the world, with some microbial populations now absent from industrialised populations (104). Indeed, there is evidence that immunologically relevant gut microbes such as *Prevotella copri*, *Eubacterium rectale*, and *Bifidobacterium longum* codiversified with humans, but it is not known how these variations in functional capacity might impact AD risk or severity in different populations (105). Population specific dietary habits can indirectly influence immune responses via the gut microbiota, as microbial fermentation generates metabolites with immune modulatory effects (106). Consumption of a higher diversity of fruits, vegetables and fermented foods were associated with a reduced risk of atopic disorders and asthma in children, potentially mediated in part by microbial-derived butyrate and propionate (107). However, the specific plant-based substrates (e.g., fiber type, fatty acids, polyphenols) that are responsible for these positive associations are unknown. It is hypothesized that the transition to reduced diversity, low-quality, highly processed, high-energy diets typical of urban or industralised populations have altered the metacommunity, its processes that underpin assembly and activity of the human microbiome and, consequently, increased risk of inappropriate and uncontrolled immune responses that damage host tissues and function (108). Population specific dietary differences may underpin many of the microbial changes in AD, but these links remain to be proven.

The skin microbiome is also an important component in AD pathogenesis (44). Pathogenic organisms such as *Staphylococcus aureus* and certain strains of *Malassezzia species* have been associated with AD and disease severity. Lifestyle factors, UV exposure, climate, age, gender, ethnicity and skin site influence the composition of the skin microbiome (109). Differences in skin microbiome composition in AD as it relates to different ethnicities and different regions that may have distinct microbial exposures have not been well documented. Due to the close relationship and bidirectional interaction between host immunity and skin microbiome, it is plausible that differences may exist. Single nucleotide polymorphisms in human beta defensins (hβDs) have been described in different ethnicities including African ethnicity (110). This may account for differences in colonization with pathogenic organisms as hβDs are important host antimicrobial peptides that are first line innate immune responses (80, 82). *S. aureus* genotyping showed differential distribution in different American ethnicities. *S. aureus* strains lacking the virulence factor gene encoding Toxic Shock Syndrome Toxin 1 (TSST-1) were detected in African Americans with AD compared to European Americans and Mexican Americans with AD (86). This may be a function of host immunity. Significant temporal shifts in *S. aureus* superantigen prevalence were also notable possibly in keeping with *S. aureus* strains cycling through the population approximately every ten years (111). The *S. aureus* genes *lukE*, *lukD*, *splA*, *splB*, *ssl8*, and *sasG* were more frequent in isolates from AD patients compared to controls in one study, but it's unknown if these molecular signatures are also present in isolates from African populations (112). Given these considerations it would be valuable to monitor *S. aureus* profiles in AD patients in different regions.

### Health disparities

Several studies have shown the association of structural racism and disproportionally high allergy prevalence in marginalized communities. In a Detroit based study in a diverse demographic of children, African American children had significantly higher allergic outcomes such as IgE sensitization to at least one food/aeroallergen skin prick test; elevated serum IgE levels; presence of atopic dermatitis and reported symptoms of wheeze compared to European Americans (113). In a study of African American paediatric AD, residential segregation as a marker of structural racism was associated with AD severity (114), which is not dissimilar to observation in South Africa, where high asthma rates were documented in marginalized communities living in heavily industrialized settings in keeping with apartheid spatial planning (115). Notably, in this study, estimated particulate matter concentrations were more than 6 times above the USA National Ambient air quality standard. Although AD was not assessed as an outcome, given the shared pathogenic environmental risk factors it is plausible that the skin could be similarly affected.

### Future perspectives

In addition to systemic immunosuppressive drugs such as methotrexate, azathioprine, cyclosporin used in moderate to severe disease, novel therapies may be useful in severe disease forms and where there are multiple comorbid allergic conditions. Recent advances in biologics for AD have ushered in the era of precision medicine owing to a more granular understanding of immune mechanisms of disease. Modulation of appropriately targeted immune mechanisms relevant to specific patients holds the most promise for AD therapies for all AD sufferers. Indeed, African populations may stand to benefit the most from precision medicine particularly in clinically heterogenous conditions like AD as Africans are the most genetically diverse population in the world (116).

In addition, within the context of pharmacogenetics biomedical variation in drug response in African populations have also been documented (116). Under the current state-of-the-art, precision medicine efforts, particularly the burgeoning use of biologics may be less well-targeted in individuals of African ancestry, as they are not well represented in the experimental data and clinical studies underlying these initiatives. There is a growing appreciation of population/ethnicity-specific disease phenotypes and corresponding immune endotypes that may additionally be unique to certain geographies and early life exposures.

Collectively this data suggests that the role of genetic background generally needs careful consideration in terms of clinical presentation of disease, diagnostic criteria, disease mechanisms and indeed treatment responses and this consideration is relevant in AD and allergic disease.

Unprecedented urbanization and urban migration are ongoing throughout the African continent and will represent the fastest urban growth rate in the world. It is estimated that the population of Africa will double by the year 2050 with two thirds of this growth concentrated in urban areas. It has been noted that 70 per cent of Africans live in urban informal settlements (117). Therefore, it will be important to study AD and allergy development as well as healthy immune development in the African urban and rural settings; to document the influence of local and specific environmental exposures, identify interventions that may mitigate against adverse health outcomes related to living environment and anticipate future healthcare needs. In addition, clarifying the contribution of genetic and epigenetic susceptibility, immune responses, socio-economic status, environmental factors, and their collective interactions to account for the disparate observations in African populations is needed.
